# LCTree-Based Approach for Mining Frequent Items in Real-Time

**DOI:** 10.1155/2022/7430106

**Published:** 2022-10-14

**Authors:** Jiashun Chen, Jianjing Chen, Zhaoman Zhong, Hao Zhang, Mehmed Kantardzic

**Affiliations:** ^1^School of Computer Engineering, Jiangsu Ocean University, Lianyungang, Jiangsu, China; ^2^Lianyungang Huajie Senior High School, Lianyungang, Jiangsu, China; ^3^School of Mathematics and Information Engineering, Lianyungang Normal College, Lianyungang, Jiangsu, China; ^4^J. B Speed School of Engineering, University of Louisville, Louisville, KY, USA

## Abstract

With the increase of real-time stream data, knowledge discovery from stream data becomes more and more important, which requires an efficient data structure to store transactions and scan sliding windows once to discover frequent itemsets. We present a new method named Linking Compact Tree (LCTree). We designed an algorithm by using an improved data structure to create objective tree, which can find frequent itemsets with linear complexity. Secondly, we can merge items in sliding windows by one scan with Head Linking List data structure. Third, by implementing data structure of Tail Linking List, we can locate the obsolete nodes and remove them easily. Finally, LCTree is able to find all exact frequent items in data stream with reduced time and space complexity by using such a linear data structure. Experiments on datasets with different sizes and types were conducted to compare the proposed LCTree technique with well-known frequent item mining methods including Cantree, FP-tree, DSTree, CPSTree, and Gtree. The results of experiments show presented algorithm has better performance than other methods, and also confirm that it is a promising solution for detecting frequent item sets in real time applications.

## 1. Introduction

With the development of information technology, such as smart mobile phone, sensor networks, fraud detection systems, and stock market systems, the amount of ubiquitous data is exploding. Different types of data can be classified into two main categories:

Static data, for which is usually in the form of transactional database, doesn't change once being stored. Static data [1–3] can be formalized as following: Let *ℕ*={i_1_, i_2_, i_3_ ⋯ i_*n*_} be a set of literals, called items. A transaction *T*with *m* items is denoted by{x_1_, x_2_, ⋯, x_*m*_}, such that *T*⊆*ℕ*. The data-base *𝔻𝔹* is a set of all static transactions.

- Dynamic data [1], a data stream *T* (also called transaction set *T*) consists of a sequence of *m* items drawn from a universe of items *ℕ*. These data items in dynamic transactions changes in time, and often with a high speed. Main properties of data stream are continuity and unboundedness.

Data mining methods for determining frequent itemsets can be classified, based on the type of data they analyzed, as (i) static data mining [4–6] and (ii) dynamic data mining (also called real-time or stream data mining) [7–9] The former is likely to dispose of transaction dataset in the database, and often needs to scan database more than once. Although a disadvantage, algorithms dealing with static data have much less strict requirements on space and time complexity than those with dynamic data. There are several reasons why methods of dynamic data mining are far more demanding:

- Because data streams continuously produce a large amount of data, it is theoretically impossible to store infinite data streams in available computer memory space. How to handle infinite data streams is a challenge, which is not emphasized in traditional static data mining techniques.
- Traditional mining methods for static data sets usually read the database several times, and mining results are obtained with non-linear complexity algorithms (sometimes polynomial, sometimes even more complex). These approaches aren't designed for streaming data where reading is enabled only once.
- Data streams are changing in real-time, and some infrequent items at a current time are becoming frequent later. These changes should be detected by streaming data mining techniques by generating dynamic models adjusted to the data stream changes.

Streaming transactions, continuously coming in time with dynamically changing frequent items, require efficient mining methods with respect to memory space, and close to linear time complexity.

The rest of the paper is organized as follows. Requirements and basic principles of the proposed approach are given in Section 2. A review of other approaches and applied frequent mining techniques are also given in Section 2. Details of LCTree implementation using head-linked-list data structure is given in Section 3. Experimental analysis with synthetic and real-world data sets is presented in Section 4, where a comparison of the proposed LCTree approach with recently developed techniques: Cantree (Canonical order Tree), FPTree (Frequent Pattern-growth), DSTree (Data Stream Tree), CPSTree (Compact Pattern Stream tree), and GTree (Group Tree) is discussed. The text is finalized with conclusions in Section 5 and references.

## 2. Related Work

Data stream mining is an important research field because of its wide spread of applications including mining medical and big data [10–12] trajectory data stream analysis [13], sensor data mining [14, 15] stock market prediction [16, 17] network traffic data analysis [18, 19].

Streaming data mining includes techniques for frequent item set detection, where the process is adjusted because of dynamics in data streams [20–22]. The main problem with mining frequent items in data streams is to find a compatible model for dynamically storing the data and, in parallel, applying all operations for determining frequent itemsets in efficient time. Generally speaking, there are three major stream processing approaches:

1. Landmark window model [23–25].
2. Damped window model [26–28] and
3. Sliding window model [29–32].

The landmark is defined as a specific timestamp. The landmark window approach starts counting frequent itemsets from a given time point and ends at the current time [23]. The importance of each transaction, with corresponding items in window, is the same independently of arrival time. The Damped window model is called the time-fading window model [33, 34]. It assigns a weight value for each transaction, and the weights are decreasing over time. The assumption is that old transactions and corresponding items are less important and they have a lower weight than the recent ones. Finally, the sliding window model requires that the user defines a size of the window [35, 36], and a fixed-length window slides over time. Set of transactions in the sliding window is treated as a unit. This model concentrates on the recent transactions in fixed size window to find frequent itemsets. The “fixed” window does not mean that window cannot vary in time, but it means that window is fixed at least for some time period, and it can be changed at a later period of time.

After the first algorithm for mining frequent itemsets in a stream has been presented [23] the real-time data mining becomes an important research field and many new methods [37–40] were developed for data stream mining. This review of a related work is concentrated on several recent algorithms for mining frequent itemsets; it includes FP-tree, Cantree, DSTree, CPStree, and Cantree-Gtree approaches.

FP-tree is regarded as an extension of the prefixed-tree technique [41], which is used to mine the completed set of frequent patterns by pattern fragment growth with the divide-and-conquer method [5] FP-tree was applied in variety of applications, and it is a basis for many extensions of the algorithm [42–44]. In the process of construction FP-tree, items are arranged in some way so that more frequently occurring items have better chances to share items than less frequent ones. A partitioning-based-divide-conquer method is taken by FP-tree, which can reduce the size of conditional patterns and corresponding conditional FP-tree. In the worst case, many algorithms that adopt FP-tree require scanning the entire database twice to update/rebuild the FP-tree. Updating may cause swapping (via the bubble sort), splitting, and/or merging of tree nodes. All these modifications require more time and space, which usually can't be performed in real-time.

Cantree [45] is an incremental algorithm for mining patterns using an expanded sliding window approach. The main merit of Cantree is that two ideas from FP-growth and Apriori were combined in the process of projection tree construction for mining frequent itemsets. When the database is updated, Cantree didn't scan the whole database again, and adjust, merge, and/or split tree nodes during maintenance, such as FP-tree which brings another problem that Cantree will keep all nodes in memory. The approach leads to increased memory utilization, and therefore Cantree isn't good at coping with real-time data.

DSTree [46] develops a tree structure to capture and maintain relevant items found in the data streams. DSTree maintains a list of frequency counts for each node to record the frequency of items which is from different sliding windows. This data structure to some extent cut down overheads of nodes inserting or removing and this is the main difference between FP-tree and Cantree algorithms. However, there is no need to keep such a frequency count list for the same node, because the same node in each transaction has the same meaning.

Cantree-Gtree [47] takes two steps to find frequent itemsets: one is to construct the base tree, and the other is using base tree to produce Gtree. Cantree-Gtree can find all frequent itemsets by using list data structure. At first Cantree-Gtree looks for the same node in a table, and then travel Cantree to find all sub-trees, and by this means they formed Gtree. In the process of construction Gtree, the algorithm needs to maintain a list for each node. The next required step for Cantree-Gtree is a construction of a projection tree, which needs to scan memory again. The algorithm maintains a lot of auxiliary lists for Gtree nodes, which will occupy plenty of extra memory. Lists play an important role in all the procedure. The support of no de_x_ is computed by adding all supports of the same no de_x_ in the list_x_. This computing and searching process is complicated. In order to find frequent itemsets, Cantree-Gtree [47] deleted nodes in the sliding-window according to supports, and then traversed the Gtree to unite the itemsets. The union of all itemsets will define final frequent itemset. In this process, nodes in the sliding-window are modified, which affects the procedure of deleting old nodes. The mining phase is time-inefficient because of the structure of the Gtree.

DSTree's method is more efficient than Cantree-Gtree in part, because Cantree-Gtree needs to maintain many lists to shift frequency of each node, just like a queue. It increases memory consumption, which is incompatible with the main expectations of data stream analysis. Data stream mining requires minimizing the usage of time and space memory as much as possible.

Cantree needs to scan database only once because all nodes are sorted by canonical order. This property also makes transactions to be easily added to the CanTree without any extensive searches for merging paths, which is different from FP-Tree and CATStree (each of them needs to scan database twice, and requires to split and merge node in the construction process).

As a kind of incremental mining algorithm, Cantree grows very fast with the coming data stream. Cantree-Gtree [47] improved Cantree. First, in Cantree-Gtree, data structure of the node includes two fields which are value and support, and excludes the node number, which is redundant for the Cantree construction and saves capacity; Secondly, in Cantree-Gtree, iTable and lTable are maintained, and nodes in iTable is used to form many lists which sever construction Gtree and find frequent itemsets, while lTable is used to remove the oldest transactions by traversing from top to bottom. In order to improve efficiency of algorithm, they represent list of candidate Gnodes (GTree Node) with two-dimensional list during the process of construction Gtree. This is the method of trading space for time. Because of rapid growth, Cantree suits more to mine static data. Both of them need more time and capacity.

To reduce the time complexity and space complexity effectively, we eliminate iTable and lTable structure, which is useless in LCTree. Instead, we present two linear linking lists, one is head list and the other is tail list. The two lists replace a large number of lists that are generated by iTable and it is necessary for limited memory capacity.

LCTree presented in this study needs to neither create a list nor delete the node from tree. Rather we reduce the frequent count of node by maintaining linking. Because some nodes are the same in transactions, we only amend the frequency count instead of deleting the node and shifting the frequency lists. Only sometimes we may need to add new one and delete the oldest when the coming nodes do not exist in the LCTree. Because our goal is to search for frequent itemsets, all items in the sliding window must exist within the same nodes which will save inserting and deleting time consumption. The worst case is each new batch needs to be added and each the old batch needs to be deleted(?). If this case is true, that all batches have no frequent itemsets which makes no sense.

## 3. LCTree: Data Structure, Construction and Mining

**Figure 1:**
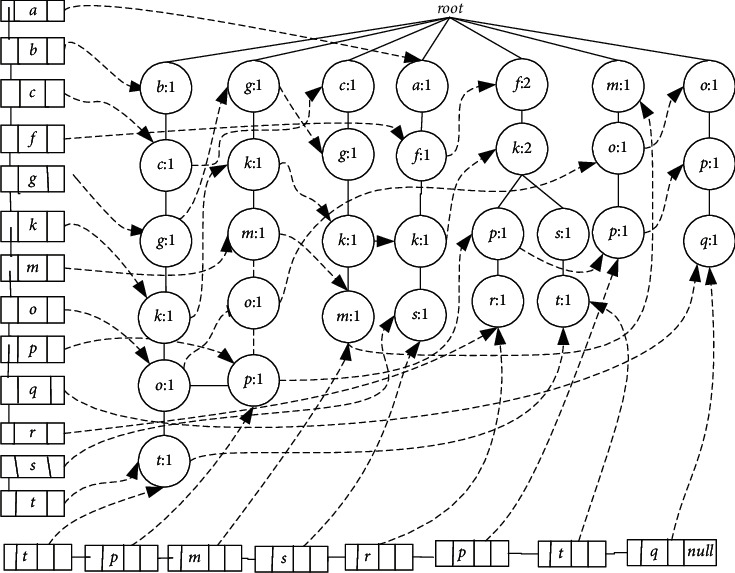
Cantree with headlinkedlist and tailinkedlist.

Our work is based on Cantree-Gtree approach, and comparing with linking cantree (see Figure 1) implementation in Leung's study [45]and Kim's study [47] we developed a method with several improvements comparing cantree and Gtree approaches. Like Cantree and Cantree-Gtree, the improved algorithm in this paper tries to find frequent itemset in a sliding window. The data streams are regarded as a serial of transaction, where a certain amount of transactions are defined in a sliding window.

### 3.1. Main Characteristics of LCTree

**Figure 2:**
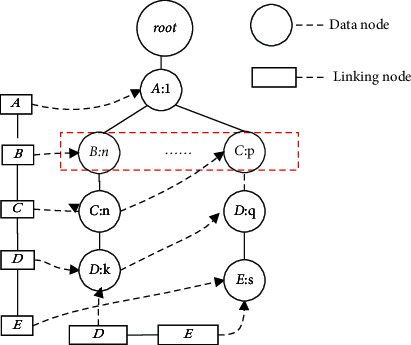
The characteristics of LCTree.

LCTree is different from previously used tree structures in frequent itemsets mining: FP-tree [5] Cantree [45, 48], CPSTree (Compact Pattern Stream tree) [49], and Cantree-Gtree(Gtree) [47]. LCTree adopts linking to construct tree and merge the nodes, and main characteristics of LCTree are summed up as following:

- Efficient head linking list In LCTree, a head linking list data structure is proposed. Unlike previous approaches [5, 47, 49], the proposed algorithm finds the inserting place easily with the linking table data structure, which is convenient to mine frequent itemsets. In order to find inserting place, scanning whole trees is necessary in algorithms of Cantree [45], CPSTree [49] and Cantree-Gtree [47], which is inefficient. Though FP-tree [5] also has linking, which seems similar to LCTree, the linking in FP-tree is only used to mine frequent itemsets, instead of coping with nodes manipulation such as merging, searching and so on. For example (see Figure 2), no de C : n is needed to find appropriate place to insert, we can directly find the hea d C according to head linking list, not searching from the root.
- Minority transactions scan The former [5] algorithms [45, 47, 49] need scan the path from the root to leaves to store all itemsets transactions in appropriate places. LCTree starts from a given node to search for appropriate place to store the transactions, rather than from top or from bottom. LCTree only needs to visit a small portion of nodes, which decreases LCTree time complexity. For example, if we find a place to insertno de x in Figure 2, we don't need to scan all data from no de b:n to no de c : n, and there will be a lot of nodes between no de b : n and no de c : n. We just scan nodes in the head linking list.
- Low cost of construction Cantree and CPStree need to traverse the tree from bottom to top or vice versa, when they build projection-tree or conditional-tree with FP-growth algorithm. Whatever way is taken, it is inevitable for construction projection-tree to require high cost to visit each node. Even in Cantree-Gtree [5, 47], where he cost is less than the other algorithm, it still traverses nodes from top to bottom. We use searching algorithm in LCTree to merge nodes, which will reduce the cost of time and space on the basis of head linking table, instead of by using traverse way. From Figure 2, we know the same node can be merged into one node, which reduces time and complexity.
- Efficient removing old transactions in Cantree-Gtree, a data structure named ltable is maintained. When they remove an old transaction from Cantree, they need to look for nodes information in ltable. After removing the oldest transactions, the Cantree-Gtree will be adjusted from the last node to its root to keep in order. Removing old nodes in LCTree will become easier. In LCTree, we create a table named tail linking, which can help find old transactions easily. There is a need to sort and merge the path node after removing the oldest transactions. For example, if we remove node *d* : *n* in Figure 2, we only browse tail link list to find node *d* : *k* and adjust the value of node no de d : k to (*k* − 1), which is convenient. We don't need to adjust the whole sub-tree nodes, which shows that algorithm in this paper is efficient.
- Linear linking data structure Cantree-Gtree [47] constructs the projection-tree by using a two-dimensional list (a list of data items, and a list of candidate gNodes for each item), which is similar to combining shell sort with binary search algorithm. The authors reported that such a method can decrease the cost of the insertion operation, but will consume more memory space. It is a thought of sacrificing memory for speed. For data stream, the algorithm tries to decrease time and capacity consumption, that is, newly generated transactions should be processed in less than a fixed duration and limited capacity to produce the up-to-date analysis result of a data stream. The presented LCTree can satisfy these requirements. We maintain a linear changeable linking list, by which inserting nodes will consume less cost of space and time. From Figure 2, we know that if we insert no de d : k into tree, we only need to find node *d* : *k* and increase the value by 1. If the no de d : k is not in the head link list, we just insert node *d* : k into the tree in appropriate place. The whole process of searching for and inserting node *d* : *k* is linear.

### 3.2. Data Structure of LCTree

In order to describe the LCTree, some formal definitions should be introduced at first.

To cope with frequent items in data stream, it is necessary to introduce basic formalisms about frequent itemsets. Let T={*x*_1_, *x*_2_, ⋯, *x*_*m*_} be a set of *m* items, and DB={*t*_1_, *t*_2_, ⋯*t*_*n*_}is a transaction database. Let ‖DB‖ be the number of transactions in database, while *T*_*x*_is a transaction including itemset *x*. User defines a threshold *θ*(0 ≤ *θ* ≤ 1) as a support for itemset (*x*), and it represents a quotient between the number of transactions contain itemset *x* defined as num (T_x_) and the total number of transactions in the database, namelySup(x)=num(T_x_)/ ‖DB‖. An itemset *x* is called frequent if Sup(x) ≥ *θ*. Based on these introductory concepts, we may introduce definition of LCTree.

**Definition 1 .**

**Figure 3:**
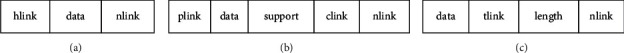
Data structure of head list, node and tail list (a) headlinkedlist (b) LCTree node (c) taillinkedlist.

Headlinklist is defined as a linking list of nodes which are from the sliding window. All nodes in headlinklist are different from each other. They are linked by hlink pointer (see Figure 3). Nodes that linked by nlink pointer have the same data. If data in headlinklist is *x*, and then we name it as **headlinkedlist**_**x**_.

**Definition 2 .**

Taillinklist is also defined as a linking list of nodes which are from last node of each transaction in the sliding window. All last nodes of transactions are linked by nlink pointer, and each last node is linked by tlink pointer.

**Definition 3 .**

**L** **C** **T** **r** **e** **e** **n** **o** **d** **e**_**x**_ is a tree node that has data *x*. in a tree, there will be the same item, and so its name is the same. But there is no effect on finding frequent itemsets, because all of the same nodes are linked by pointer.

**Definition 4 .**

LCTree is a tree with headlinklist and taillinklist, where headlinklist has the same node of all transactions in sliding window and taillinklist has all the last nodes in each transaction. All items in headlinklist are linked by hlink pointer.

The data structure of head list, tree node and tail list are defined as Figure 3. The reason that we use linking lists instead of lists is to avoid sorting the item before being put into headlinklist. Headlinklist has been defined three parts, hlink, data and nlink. Hlink points to the next head node, and nlink points to the next node which has the same value to head node in the LCTree. Data in headlinklist stores item value. There is no need to order the items value before entering the headlinklist. Figure 3 shows data structure of head list, node and tail list.  Figure 3(a) shows the structure of headlinkedlist, while Figure 3(b) is the LCTree node structure, and taillinkedlist is described in Figure 3(c).

The five components included in LCTreenode are plink, data, support, clink, and nlink. Pointer plink is used to find the parent and data is the item(?). Support is a recorded number of the item used to find frequent itemsets. Poniter clink and nlink in the LCTree mode point to child and next nodes with the same item to headlinklist respectively. The meaning of nlink in LCTreenode is the same to nlink in headlinklist. LCTreenode data structure is shown in Figure 3(b). Structure nlink in taillinkedlist has the same function as nlink in headlinklist and LCTreenode. Tlink in taillinklist points to all tail nodes in sliding windows. It is a fact that tailnodes is not just leaf nodes of LCTree. It could be ordinal tree nodes. With the help of tlink, traversing from leaf to root can determine one transaction. Time complexity of this operation is linear with the length of transaction. Tlink plays a significant role to remove the oldest transactions in sliding windows, which will be illustrated in later section.

### 3.3. LCTree Construction Algorithm

In this part, we will introduce the construction process of LCTree. In Cantree-Gtree algorithm, Gtree is a projection-tree, which is used to find frequent itemsets and remove the oldest transactions. Gtree is the basis of all other operations. Gtree is created from Cantree, which means that Cantree is constructed first and then Gtree is built. From the construction process of Gtree, we know the transactions in the window need to be scanned twice: the first is to build Cantree; the second is to build Gtree, which will consume extra time. Unlike the Cantree-Gtree, constructing LCTree do not need to scan transactions twice. LCTree doesn't need to build base tree nor projection tree, and so there is no need for extra steps to create LCTree, which is one of differences between LCTree and other main data stream algorithms such as [5, 45, 47, 49]. Just for this reason, LCTree can save time.

**Figure 4:**
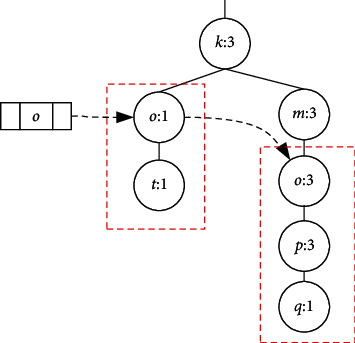
Search for the max branches for LCtree.

LCTree is initialized as an empty root node. The process of construction is to insert items into LCTree. In Algorithm 1, there are two main steps to construct LCTree. In general, Algorithm 1 includes two steps: one is searching for node; the other is to cope with node. If one node is not in headlinklist, the algorithm inserts the node into headlinklist and link to taillinklist and headlinklist, which is completed by function insertheadlinklistH() (see Algorithm 2) and insertheadlinklistN() (see Algorithm 3) and inserttailinkedlist() (see Algorithm 3). Corresponding parameters need to be adjusted. On the contrary, instead of inserting (?), algorithms need to look for the longest branch in LCTree. Headlinklist can help accelerate this process which is realized by using function searchmaxprenode() (see Algorithm 5).  Algorithm 6 is to match two branches, and it is called by Algorithm 5. For example, when transaction *T*_8_ is inserted into LCTree, because there are two branches including nodeo, namely {*o*, *t*} and {*o*, *p*, *q*}. Function searchmaxprenode finds branch {*o*, *p*, *q*} is the maximal one (see Figure 4). The rest to do is the same as above operations, such as linking, inserting and so on. So construction LCTree is simpler than Cantree and Cantree-Gtree.

In order to explain the contents mentioned above, we will give some illustrative examples here.  Table 1 is an example of the sliding windows. In the table, it shows eight transactions, and each transaction includes different numbers of items. There are one sliding window in Table 1. All items in the same transaction are different. All items in the example are sorted in alphabetical order.

we take partial nodes from Table 1 as an example to construct Cantree with headlinklist and taillinklist (see Figure 1), so as to show that even if linking data structures in LCTree are applied in Cantree construction, it has no optimal structure to save data point. Items are from transactions T_1_ ~  T_8_ in sliding window, and they are connected by hlink pointer. In hea dl inke dl ist_*d*_, all of the same node *b* is connected by nlink pointer, so do hea dl inke dl ist_*e*_. Nlinks in taillinklist point all last nodes. It's worthwhile to note that the last nodes in taillinklist maybe have the same item. We insert it into taillinklist in the order they appear in the transactions. From Figure 1, we know that projection tree grows very fast even if the linking is used, just like FP-tree. We should use new methods to decrease the scale of the projection tree.

Compared to other trees, such as FP-tree, Cantree, Cantree-Gtree, and tire tree, LCTree is different because of the following reasons:

First, LCTree has a different way of storing node and its child. As we know, Cantree-Gtree is formed by maintaining a large number of lists. Each node has one list to put its one child. That is to say, if one node has ten children, algorithm must form ten lists for the node to store its children. If the transaction is too large, memory consumption is incredible. In LCTree, only two linking lists (headlinklist and taillinklist) are needed and all transactions are stored in them. A remarkable fact is that data stored in headlinklist are different, which can save memory capacity. Pointer nlinking in LCTree helps to search for tree node and frequent itemset (which will be illustrate in the next part), which is the main improvement of LCTree over Cantree-Gtree.

**Figure 5:**
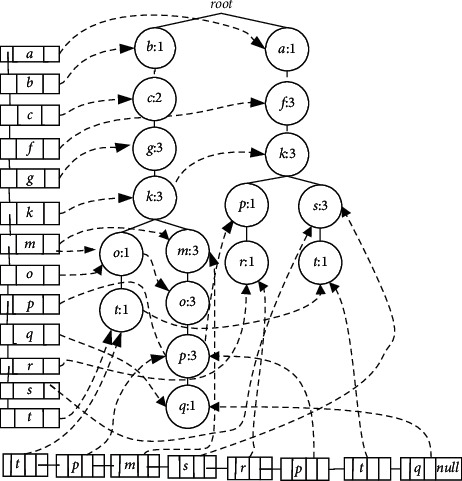
LcTree with headlinklist and taillinkedlist, formed with transcations *T*_1_∼*T*_8_.

Secondly, LCTree is used to find frequent itemsets in real-time data stream while FP-tree is wide used in static database. The main difference is in construction algorithm. Because there is no pattern match process during the process of construction, FP-growth method makes FP-tree grow fast, which will run out of memory capacity if there is a big database. While with the help of headlinklist, LCTree uses a sub-procedure to find the maximal branches, which can control the tree growth effectively. Many of the same nodes share the same branches, which is also easier to form frequent itemsets. For example, Figure 1 grows faster than Figure 5 with the same transactions. According to the FT-tree algorithm, few transactions can be merged in Figure 1, just T_4_and T_7_, which is against finding frequent itemsets.

Thirdly, some LCTree's children share the same partial branch, not whole branch as in the case of trie tree. Trie tree has a requirement that all children of one node must have the same prefixed nodes. Unlike tire tree, node in transaction matching with other node in LCTree is from the beginning of any LCTree node, not from the root. For example, in Figure 5, node o and node *m* have different prefix branches, and the prefix branch of node o is branch {*b*, *c*, *g*, *k*} while prefix branch of node *m* is branch {*g*, *k*} or branch {*c*, *g*, *k*}.

Our work is a true oriented to dynamic data, while FP-tree is efficient algorithm for static data. Our work is also different from other algorithms. We don't need to keep a list of counts for each node to insert and remove tree nodes; nevertheless, we just use two linking tables to implement this requirement, which is different from DSTree. We also don't maintain a lot of list to construct Gtree to mine frequent itemsets after constructing projection tree. In our algorithm, only one tree is needed to complete inserting and removing nodes and mining frequent itemsets, which is different from Cantree-Gtree.

### 3.4. Mining Frequent Itemsets

According to the definitions of frequent itemset, support is greater than or equal to the given threshold. We set the threshold to 3. Figure 5 is the LCTree from transaction T_1_ ~ T_8_ in Table 1. Transactions T_1_ ~ T_4_ and T_5_ ~ T_8_ belong to window SD1. We are searching for frequent itemsets from SD1 now.

To find all frequent itemsets, the overall mining process implementation is divided three steps. (1) algorithm needs to find the node from headlinklist; (2) the related node is searched in LCTree by using pointer nlink in headlinklist; (3) all frequent sub_LCTree is found. In this step, the support of related node has to be summed and then compared with the threshold. All nodes and their branches will be put into frequent itemsets, if their support is equal or greater than the threshold. For example, we get the first head node *a* from headlinklist. According to nlink in headlikedlist, we find node *a* in LCTree. All support of nodes *a* in LCTree should be summed by using nlink. In this place, nlink of node *a* is empty, that means there is no node *a* in LCTree. All supports of node *a* are less than threshold, and in this case node *a* and all his children will not be taken into account. Node *b* and *c* is similar with node *a*. Support of node *f* is greater than the threshold. Node *f* has one branch, and so we find all his children whose supports are greater than the threshold (see Table 2). Node *g* has four children whose supports are greater than the threshold. The frequency of node *g* is shown in Table 2. Node *k* has two branches which is different from the former nodes. We add all supports of node *k*whose support is six, and so it is frequent item. We will take into account all its branches. We find both branch {*k*, *p*, *r*} and {*k*, *s*, *t*} does not meet the threshold requirement, hence they will not be considered. Another branch{*k*, *m*, *o*, *p*} meet the threshold requirement, so we find frequent items of node *k* (see Table 2). The rest nodes such as nodes *m*, *o*, *p* have the same operation to the former. Support of nodes *q*, *r*, *s*, *t* is less than threshold, and so they are not listed in Table 2. This method not only gets all frequent nodes but also is easier than the algorithm in Cantree-Gtree [47].

### 3.5. How to Delete the Oldest Transactions

In the window model (see Table 1), when the window is full, the oldest transaction will be removed so as to let new transaction come in. LCTree can easily find and delete the oldest transactions with help of taillinklist structure. Nodes in taillinklist were inserted in arrival chronological order. According to taillinklist, we know the first node is the oldest one, and the last is the newest. In data structure taillinklist, each tail node records the length of this branch. When the oldest transaction needs to be deleted, the first step is to find the head pointer of taillinklist, and then traverse the transactions along tlink pointer. In this process, the support of each node is reduced by 1, so does the length of the branch.  Algorithm 7 shows the implementation steps. For example, in Figure 5 the first node in taillinklist is no de_*t*_. According to pointer tlink, no de_*t*_ can be searched in LCTree.

**Figure 6:**
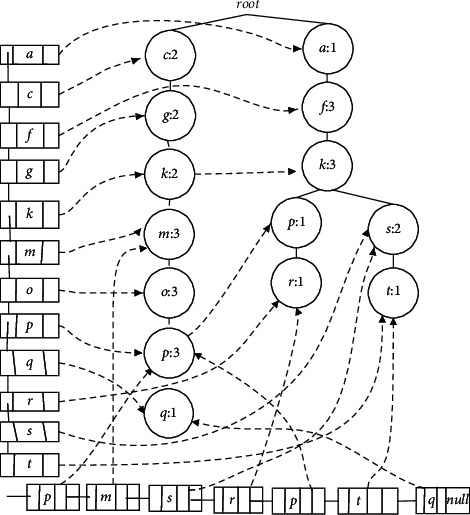
LCTree after removing transactions *T*_1_.

Support of no de_*t*_ is reduced by 1. Because support of no de_*t*_is zero, no de_*t*_ should be removed from LCTree. Relative pointers need to be adjusted. At the same time, length in taillinklist also reduces by 1. Length is not zero, each node from no de_*t*_ to no de_*b*_ will be coped with at the same operation along plink. By this way, the oldest transaction is removed. Figure 6 shows results after removing transaction, Figure 6 shows results after removing transaction.

### 3.6. Time and Space Complexity for LCTree

#### 3.6.1. Time Complexity

Time and space complexity for LCTree include two phases, namely construction LCTree and mining frequent itemsets. We analyze time complexity by proofing Theorem 1 and Theorem 2.

**Theorem 1 .**

Let *n* be the number of items, and time complexity of construction LCTree is *O*(*n*) (proof will be shown in part of time complexity)

**Proof of Theorem 1.**

Algorithm 1 is to construct LCTree. We know the number of items in each transaction is far less than the number of transaction. So the number of item in each transaction can be regarded as a constant *λ*, namely *λ* ≪ n. Searching for maximal pre-no de_*x*_ is to find the same node in LCTree. The maximal times of comparison is no more than the number of items in one transaction, which means searching scope is small. So we can set the time of comparison as a constant *δ*. In Algortihm 2, no de_*y*_is from some transaction. The number of node access is no more than the number of items in one transaction. Therefore, access rate is set as constant *θ*. The whole time complexity can be expressed by formula (1)

(1) $$\begin{array}{c} O\left(Ltree\right)=O\left(\lambda \right)+O\left(\max\;\left(\lambda ,\delta ,\theta \right)∙n\right)\text{.} \end{array}$$

In formula (1), because *λ*, *δ*, *θ* is far less than n(*λ* ≪ n, *δ* ≪ n, *θ* ≪ n), the time complexity is regarded as O(*n*).

**Theorem 2 .**

Let *n* be the number of items, and time complexity of finding frequent itemsets is O(*n*) (proof will be shown in part of time complexity)

**Proof of Theorem 2.**

In Algorithm 8, there are two while loops. By analyzing the algorithms, we know these two loops just scan the data stream once. The first loop is to find no de_x_ and the next loop is to scan all the same no de_*x*_ in LCTree. When the first loop is completed, the data stream is scanned only once. So the two loops are linear. Assume the number of node in headlinklist is *a*, and the number of the same node is *b*. According Algorithm 8, the maximal length is (*a*+*b*), and so the cost of scan is proportional to O(*a*+*b*). To summarize, time complexity of finding partial frequent itemset is O(*n*). In Algorithm 7, there is only one loop. So time complexity is O(*n*). Time complexity of finding frequent timesheets is shown by formula (2).

(2) $$\begin{array}{c} O\left(FreqItemsets\right)=O\left(n\right)+O\left(n\right)\text{.} \end{array}$$

From formula (2), we know time complexity of finding frequent itemsets is O(n).

#### 3.6.2. Space Complexity

With the help of headlinklist, many nodes that are the same can be merged. Each branch has maximal nodes that are the same. LCTree is growing much slower than Gtree [47]and other algorithms [5, 45, 46, 49], and so the number of nodes in memory produced by LCTree is far less than by theirs. For example, in Figure 1, branches {cgko} and {cgkm} have the same branch {cgk}, however, other algorithms can't merge the same parts, and so tree grows fast. Of course, a large number of memory spaces are consumed. In contrast, LCTree is constructed using only two lists in Algorithm 1. Algorithm 8 removes branches and nodes whose support is less than threshold during the process of scanning. These two algorithms have a significant reduction in memory consumption because nodes were removed before forming extra list, which means there is no necessary to create extra lists to store a large number nodes. Whereas Gtree needs to form extra list for each nodes and then removes some lists by pruning. Thus space complexity will be cut down sharply. This space complexity difference will be seen in the Experiments and Analysis section below.

## 4. Experiments and Analysis

To evaluate the proposed LCTree method, we have conducted experiments on synthetic dataset T10I4D100K and T40I10D100K [50], and real dataset mushrooms [51], connect-four (“connect-four,” n.d.) and chess [52]. Synthetic datasets are obtained from the IBM Quest Data generator. The other three datasets are from UCI. All experiments are conducted on a personal computer with Intel i7 CPU, 8G RAM, Windows 7 64bit OS, and C++ programming environment.

### 4.1. Datasets and Parameters Setting

Information about the five dataset is listed in Table 3. Mushroom and chess datasets are smaller database, containing 8,124 and 3,196 transactions, respectively, while connect four is a very large and dense database and produces many long and frequent itemsets, even for high support values. T10I4D100K and T40I10D100K are synthetic dataset, whose transactions are the same but lengths of transactions are different. The average length of transactions (ALT) in T40I10D100K is much longer than T10I4D100K. The parameter (ALT) can determine which algorithm has better performance when they deal with large and complex datasets. The character “*T*,”“*I*” and “*D*” in the name of two datasets represent the average transaction size, average maximal potentially frequent patterns, and the number of transactions, respectively.

The last column in Table 3 is the window and transaction size. The expression “*m∗n*” in window size means the number of batches (*m*) multiples the number of transactions (*n*) per windows. We set the different sizes according to the character of dataset. The column of average distinct items means that each transaction includes the number of distinct items. The larger the value is, the more distinct items are per transaction, and the lower the value of “*n*.” On the contrary, if the value of average distinct items is low, the “*n*” value should be high. The value of “*m*” is proportion to the number of transactions. For example, if value of average distinct items in dataset mushrooms and chess are high, then the size of each transaction is low. Additionally, because the number of transaction in two datasets are small, the number of windows is also low. When we look at the other three datasets, the size of transaction should be high because of low value of average distinct items. If the value of “*n*”is set as low, perhaps it does not include distinct items in transactions(?). It is insignificance for data mining and cannot test the performance of all algorithms(?). We also implemented methods in current papers [45, 46, 49] and obtained parameters of size. However, our method is different from theirs, especialy for computing average distinct items. We set the size of the window as shown in Table 3.

### 4.2. Experimental Results

The proposed algorithm LCTree is compared with Cantree, DSTree, CPSTree, Cantree-Gtree, and FP-tree. These algorithms are relevant and have good performance for data mining including streaming data mining. The criteria for performance evaluation are as follows: runtime, memory usage on changing support settings, and scalability. Such criteria are the standards widely used in sliding window-based approaches for streaming data mining.

#### 4.2.1. Runtime Comparison

**Figure 7:**
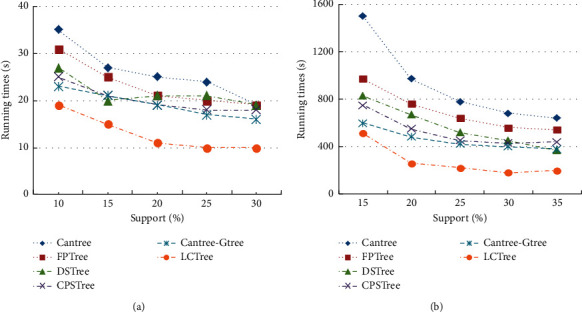
Runtime comparison on T10I4D100K and T40I10D100K for cantree, FP-tree, DSTree, CPSTree, Gtree and LCTree. (a) T10I4D100K. (b) T40I10D100K.

We tested all algorithm using two synthetic datasets, and Figure 7 presents different characteristics of a runtime for six tested algorithms. The results show a big disparity by running on two datasets. Each algorithm has a far longer runtime on T40I10D100K than on T10I4D100K. Although the size of two datasets is the same, the dataset structures are different. The average length of T40I10D100K is greater than T40I10D100K. Much more time is required to construct a projection tree for every algorithm on T40I10D100K with the same window size. The gaps between algorithms are also more apparent on T40I10D100K, which shows that six algorithms have their own special features, when they were tested with the complicated dataset. As shown in Figure 7(b), except for LCTree, the other algorithms showed obvious deficiency, especially for Cantree. Cantree needs more time not only in constructing projection tree but also in maintenance cost. FP-tree [5], DSTree [46], CPStree [49], and Gtree [47]are based on Cantree and use FP-growth [53] method to construct projection tree. So the runtime of the four algorithms is closed, as presented in Figure 7. However, there are some gaps between them. FP-tree need to scan database twice to find frequent itemsets, and so it will require more time, which can be seen in Figure 7. The structure of DSTree is slightly different from FP-tree. DSTree maintains a list of support and captures fluent data in the sliding window by scanning dataset once. In fact, the base tree of DSTree is Cantree. DSTree uses the FP-growth to mine frequent itemsets. So the runtime of these two algorithms is similar. We can find this from Figure 7. CPSTree takes FP-tree as projection tree and finds frequent itemsets easily without scanning twice. CPSTree has better performance which is shown in Figure 7. Gtree also employs the Cantree as base tree and construct Gtree as projection tree. On the process of building projection tree, Gtree deletes infrequent itemsets by combining several sub-trees. Runtime of Gtree is less than CPSTree. Figure 7 also clearly manifests that LCTree outperforms the other trees according to runtime with high and low support values. Because the process of constructing LCTree is different from any other method, LCTree does not need base tree and therefore combines two steps into one. Hence all operations can be carried out in one tree, which can save much time. The LCTree uses linking to find the adequate ranches for itemsets, which is essentially different from linking in FP-tree [5] that has been elaborated above. The old items in LCTree are easy to find and they are removed by using tail linking structure. This also can reduce the time consumption. All these analyses are the reason why LCTree has the best performance among all algorithms.

The previous results on synthetic dataset show that LCTree has the lowest runtime. Someone may wonder what performance on real datasets for all algorithms. So we also need to compare all algorithms on real datasets.

**Figure 8:**
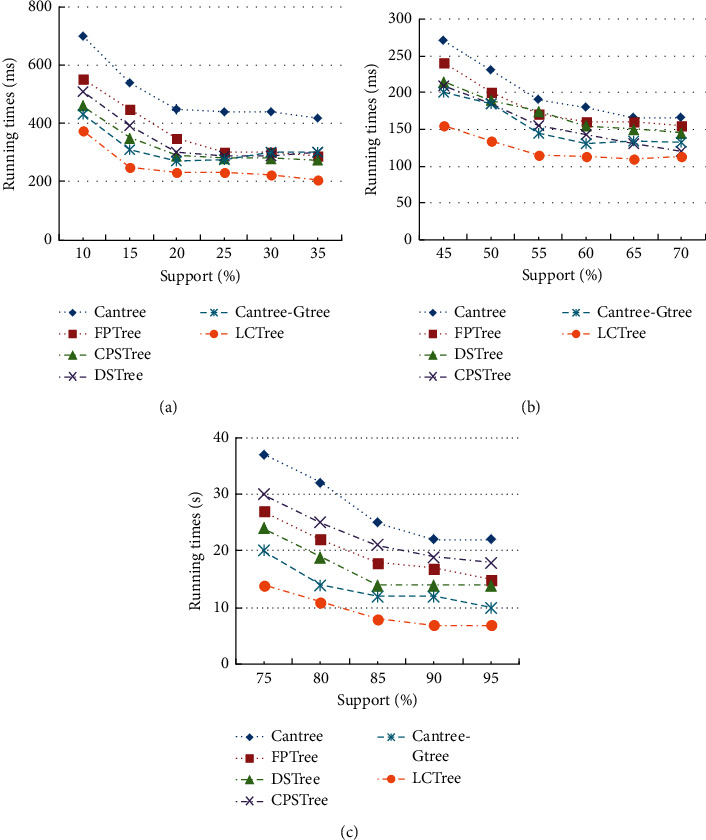
Runtime comparison on mushroom, chess and connect four for cantree, FP-tree, DSTree, CPSTree, Gtree and LCTree (a) mushroom (b) chess (c) connect four.

We tested all algorithms on two small datasets mushrooms and chess. The data size of two datasets is similar, and therefore the comparison of results is reasonable. The runtime in all figures includes time for a tree construction and frequent itemsets mining. *Y*-axis of Figures 8(a) and 8(b) shows the changing runtime of all algorithms with different support values. Experimental results of all algorithms present that runtime decreased with support rising. There is no significant difference except for Cantree and LCTree. Runtime of Cantree is greater than other algorithms. There are some reasons for these results. Cantree is a tree structure designed for incremental mining and so Cantree needs more time to construct a projection tree. The gap between LCTree and others is not obvious; however, from Figures 8(a) and 8(b) we notice that the runtime of LCTree is less than other algorithms, that is to say, on small real dataset; LCTree is superior to other algorithms. Like mushrooms and chess, connect four is also real dataset, but it includes far more data than the other two. From Figure 8(c), we see that gaps in performance between algorithms are increased when the size of the dataset becomes large. The performance of each algorithm thus is more distinguishable. Just like on the small dataset, runtime of Cantree is the longest among all algorithms, and the gap between Cantree and other algorithms is increased. The process of construction projection tree is extremely time-consuming for Cantree, when facing large data. For CPSTree [49] FP-tree [5], DSTree [46], and Gtree [47], runtime are decreased in turn. CPSTree should have better performance than any other one. However, it is the worst one among four algorithms in Figure 8(c). In Table 3, value of average distinct items for connect four is lowest among real dataset. The possible reason is that CPSTree is not suitable to cope with the connect four dataset, which can be proved by Figures 8(a) and 8(b). Results of CPSTree in Figures 8(a) and 8(b) are much better than (c), because the datasets of mushroom and chess have high value of average distinct items. LCTree showed superior performance among all algorithms because of the proven linear time complexity.

#### 4.2.2. Memory Space Requirements Comparison

**Figure 9:**
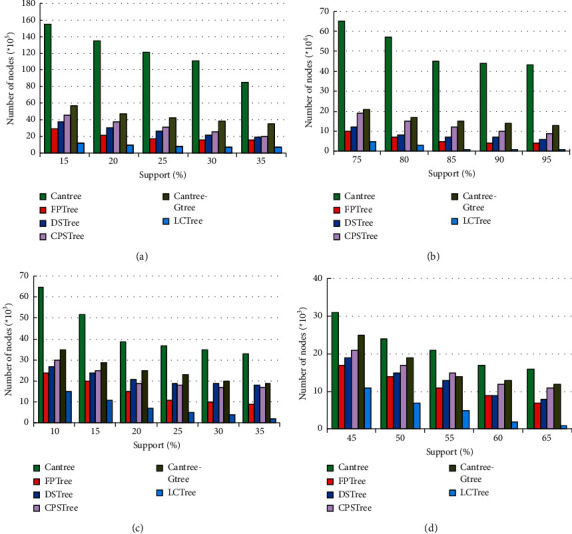
The comparison of memory overhead on T10I4D100K, connect four, mushroom and chess for Cantree, FP-tree, DSTree, CPSTree, Gtree and LCTree. (a) T10I4D100K. (b) Connect four. (c) Mushroom. (d) Chess.

Memory usage is one of the most important measures for algorithms dealing with data stream with large data volume. We evaluated algorithms according to the number of nodes generated by each algorithm, assuming that the size of nodes reflects the memory requirement of the algorithm. *X*-axis and *y*-axis in Figure 9 represent support and the number of nodes, respectively. We test the required memory by varying the support at each dataset. From the figure, we see memory footprints for all algorithms decrease with the support increasing. In all graphs, the amount of nodes created by Cantree is the largest among all algorithms on several datasets. However, there are some differences among the other four datasets. The differences between Cantree and other algorithms are increased when items in database are large. With large data volume, the sliding window of Cantree has to cope with more items. Because Cantree keeps all infrequent itemsets, it makes memory increase rapidly, a major disadvantage of the algorithm. In the other analyzed algorithms, number of nodes generated by Gtree is larger than other algorithms on all datasets. There are two reasons for these results. First, Gtree creates Cantree as based tree to construct projection tree. In this process, Gtree needs to create a lot of lists for each item to store sub-tree branches, which will influence the number of nodes in memory. Second, in order to reduce the cost of insertion operation, Gtree uses two-dimensional list to represent lists, which trades more space for less processing time. We also notice that FP-tree is slightly better than CPStree and DSTree. In essence, these three algorithms adopt the idea of pre-Tree. FP-tree is a kind of method for approximate stream mining and mines the static data, while CPStree and DSTree can get exact mining results for stream data, which explains the results in Figure 9 among the three algorithms. From Figure 9(a), FPTree has the best performance in dataset T10I4D100K among the five algorithms. When statistical analysis were performed by comparing LCTree and FPTree, LCTree appeared to save memory capacity from 42.69% to 58.42% at different support value, respectively. In Figures 9(b)∼9(c), we get similar results. LCTree saves memory capacity averaging 50.33% comparing to FPTree. No matter in the real or synthetic dataset, LCTree has the best performance. There are two causes for getting such results. First, in the process forming LCTree, we don't need too many extra lists for mining frequent itemsets. The number of lists LCTree need is far less than Gtree. Second, just like what the analysis above has shown, LCTree can find the longest braches to merger items with the help of head linking list, which is good for not only saving time for the next step of mining frequent itemsets but also significantly cutting down the requirement of memory. Experimental results are consistent with our analysis and we also conclude by comparing Figure 1 with Figure 5. The third is that LCTree has a most efficient tail linking structure, which is easy to delete the old items, but doesn't need too much operation(?).

As analyzed above, we can conclude that LCTree has a good design which makes the mining process more efficient than other algorithms on different types of datasets. Both theoretical analysis and experimental results support our conclusion.

## 5. Conclusions

LCTree is for mining frequent itemsets over data streams and is based on the sliding window model. LCTree effectively finds the appropriate branches and removes the items and also correctly searches for frequent itemsets with help of linked structure. The extensive experiments presented in this study showed that the proposed algorithm outperforms recently developed algorithms Cantree, CPSTree, FP-tree, DSTree, and Gtree in runtime, usage of memory, on both synthetic and real datasets. Compared with the five algorithms on different dataset, the runtime and capacity of LCTree is improved to some extent, which depended on supported value. These improvements may help in LCTree implementations for streaming data environment, which includes network traffic analysis, weblogs analysis, market basket analysis, and so on.

We prove the efficiency of LCTree on synthetic and real datasets. In future studies, we hope to apply them to some fields and improve performance in various fields.

## Figures and Tables

**Algorithm 1:**
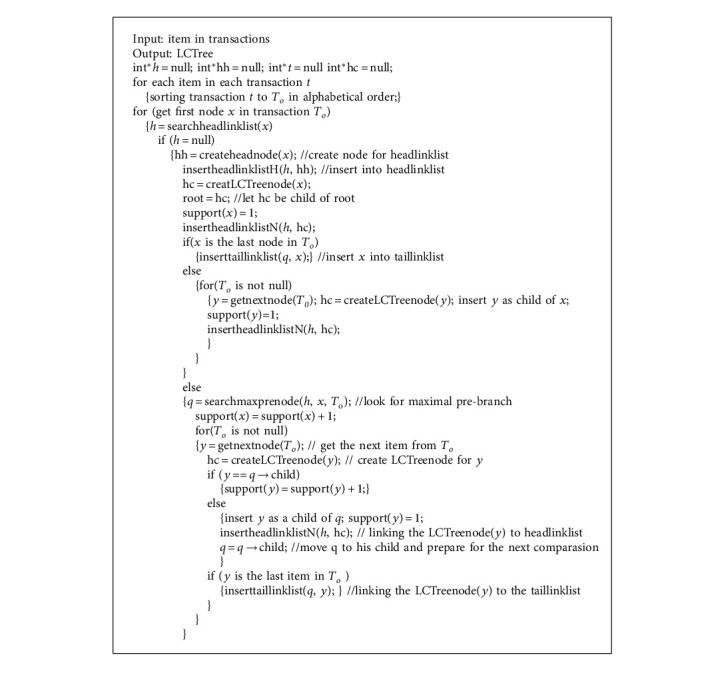
Construction LCTree algorithm.

**Algorithm 2:**
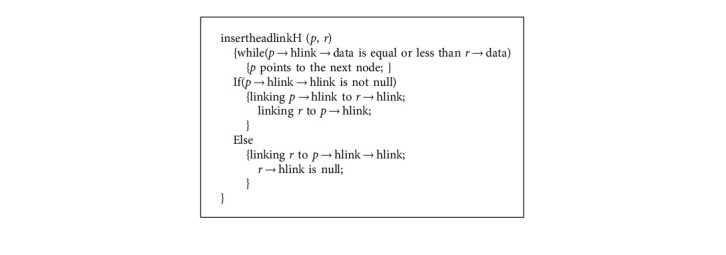
Adding headnode into headlinklist algorithm.

**Algorithm 3:**
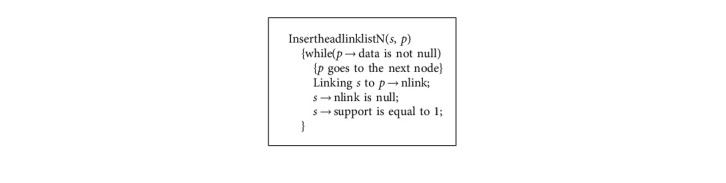
Linking LCTreenode to headlinklist algorithm.

**Algorithm 4:**
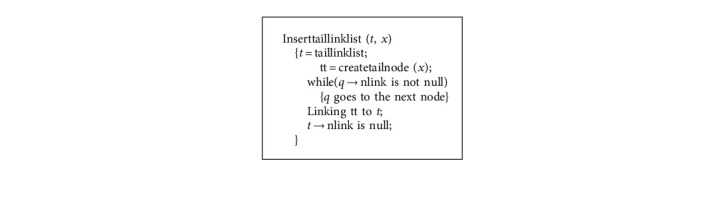
Linking LCTreenode to taillinklist algorithm.

**Algorithm 5:**
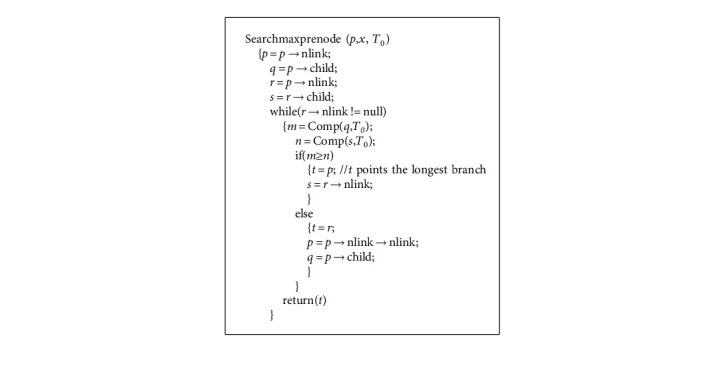
Search for the maximal branch of *x*.

**Algorithm 6:**
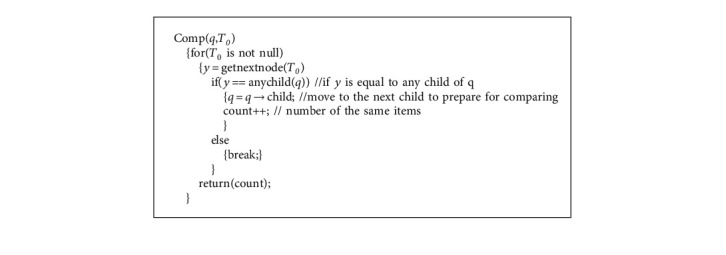
Compare two branches to find the longer branch.

**Algorithm 7:**
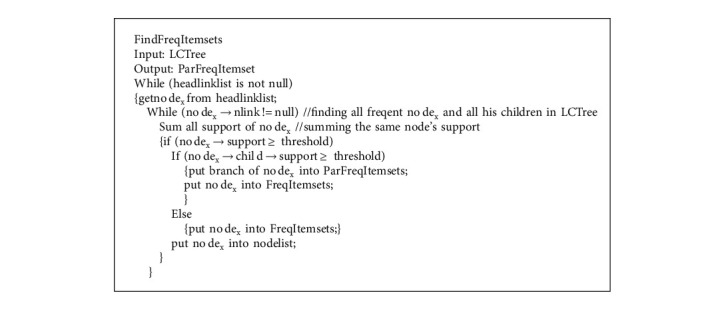
Finding all frequent itemsets.

**Algorithm 8:**
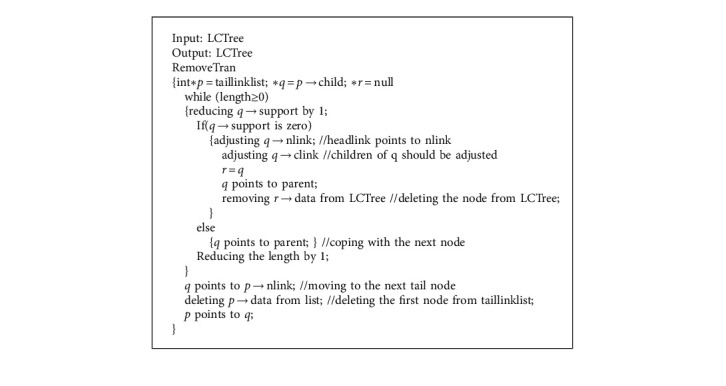
Removing the oldest transaction from LCTree.

**Table 1:** Items in sliding window.

Sliding windows	Transactions	Items
SDW1	T_1_	{*k*, *t*, *g*, *c*, *b*, *o*}
	T_2_	{*m*, *p*, *o*, *k*, *g*}
	T_3_	{*k*, *m*, *g*, *c*}
	T_4_	{*k*, *f*, *a*, *s*}
	T_5_	{*p*, *k*, *r*, *f*}
	T_6_	{*o*, *m*, *p*}
	T_7_	{*s*, *t*, *f*, *k*}
	T_8_	{*p*, *o*, *q*}

**Table 2:** Some nodes meet threshold and finding all frequent items.

Nodelist	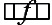	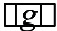				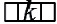			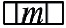		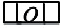	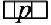
Sub_LCTree	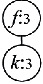	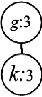	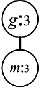	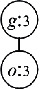	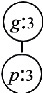	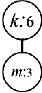	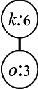	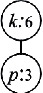	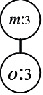	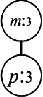	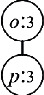	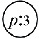

Itemsets	{*f*}{fk}	{*g*}{gk}{gm}{go}{gp}				{*k*}{km}{ko}{kp}			{*m*}{mo}{mp}		{*o*}{op}	{*p*}

**Table 3:** Parameters setting and statistical information of five datasets.

Dataset name	Relevant parameters						
	Transactions	Distinct items	Maximal length	Minimal length	Average length	Average distinct items (*∗*100%)	Windows size (*m∗n*)
Mushrooms	8124	119	23	23	23	1.46	2*∗*1K
Connect four	67557	129	42	42	42	0.19	5*∗*5K
Chess	3196	75	42	42	42	2.34	1*∗*1*K*
T10I4D100K	100000	872	29	1	10	0.87	5*∗*5K
T40I10D100K	100000	940	77	5	39	0.94	5*∗*5K

## Data Availability

The Chess and Mushrooms data used to support the findings of this study have been deposited in the UCI repository (https://archive.ics.uci.edu/ml/datasets.php).
